# Extracellular Vesicles, Influential Players of Intercellular Communication within Adult Neurogenic Niches

**DOI:** 10.3390/ijms21228819

**Published:** 2020-11-21

**Authors:** Morris Losurdo, Mariagrazia Grilli

**Affiliations:** Laboratory of Neuroplasticity, Department of Pharmaceutical Sciences, University of Piemonte Orientale, 28100 Novara, Italy; morris.losurdo@uniupo.it

**Keywords:** adult neurogenesis, extracellular vesicles, neural stem cell, neuron, astrocyte, microglia

## Abstract

Adult neurogenesis, involving the generation of functional neurons from adult neural stem cells (NSCs), occurs constitutively in discrete brain regions such as hippocampus, sub-ventricular zone (SVZ) and hypothalamus. The intrinsic structural plasticity of the neurogenic process allows the adult brain to face the continuously changing external and internal environment and requires coordinated interplay between all cell types within the specialized microenvironment of the neurogenic niche. NSC-, neuronal- and glia-derived factors, originating locally, regulate the balance between quiescence and self-renewal of NSC, their differentiation programs and the survival and integration of newborn cells. Extracellular Vesicles (EVs) are emerging as important mediators of cell-to-cell communication, representing an efficient way to transfer the biologically active cargos (nucleic acids, proteins, lipids) by which they modulate the function of the recipient cells. Current knowledge of the physiological role of EVs within adult neurogenic niches is rather limited. In this review, we will summarize and discuss EV-based cross-talk within adult neurogenic niches and postulate how EVs might play a critical role in the regulation of the neurogenic process.

## 1. Introduction

It is now commonly accepted that discrete regions of the adult mammalian brain host neural stem cells that divide in situ and give rise to new neurons, a phenomenon referred to as “adult neurogenesis”.

The two most characterized neurogenic niches are the subgranular zone (SGZ) of the hippocampal dentate gyrus (DG), a brain region in which adult neurogenesis was confirmed in humans [1] and the subventricular zone (SVZ) of the lateral ventricles, whose relevance in adult human physiology is debated. Although SGZ and SVZ neural stem cells (NSCs) and neural progenitor cells (NPCs) share many features at cellular and molecular levels, the route for adult-born neuron integration in pre-existing neuronal circuits and their ultimate outcome in the two regions are distinctive. SVZ neurogenesis involves neuroblast migration along the rostral migratory system (RMS) to the olfactory bulb (OB), where they terminally differentiate into distinct types of olfactory neurons that are mainly inhibitory neurons [2]. In the last decades, pivotal preclinical studies, especially in rodents, have contributed to the idea that the integration of adult-born olfactory neurons facilitates continuous adaptation to environmental olfactory cues [3]. Conversely, mature granule neurons that originate from SGZ NSCs are excitatory neurons that are restricted to the granule cell layer (GCL), with minimal migration. At present, newborn DG neurons are considered to be crucially involved in specific types of hippocampal-dependent learning and memory, in stress and emotional responses [4,5]. Recently, adult neurogenesis has also been identified in the hypothalamus [6,7]. Here, tanycytes, which line the walls of the infundibular recess of the third ventricle, have been suggested as putative hypothalamic NSCs since they share the characteristics of SVZ and SGZ stem cells. In this region, adult-born neurons are regarded of crucial importance for the regulation of metabolism, energy balance [8] and systemic aging [9].

Regardless of the neurogenic region and the underlying complex functions, under physiological conditions, each step of adult neurogenesis needs to be tightly controlled by both niche-derived signals and by extrinsic environmental cues, which, together, ensure appropriate rates of NSC proliferation, differentiation, migration, neurite extension and integration of newborn cells into preexisting circuits [10,11]. This extensive modulation underlies the functional plasticity that is intrinsic to the neurogenic process, by which the brain outcome can be optimized for the needs of a given environment and/or experience.

It is generally accepted that a complete understanding of brain plasticity requires consideration of glial cells in the overall picture, and adult neurogenesis intriguingly connects neuronal and glial biology. Although all types of glial cells are directly or indirectly related to this process, astrocytes and microglia take on a prominent and active role. Astrocytes provide the closest link between adult neurogenesis and glial biology. In fact, several “astroglial” properties characterize NSCs in both neurogenic zones. The additional presence of essential non-neurogenic astrocytes within adult niches is also crucial for proper neurogenic process [12]. Astrocytes—which represent the most abundant cell type of the neurogenic niche—have been largely described as key regulators of the neurogenic process [12,13,14]. In the adult niche, astrocytes physically interact with NSCs [15,16,17] and with both developmentally and adult-born granule neurons [18]. In this context, they regulate NSC proliferation, differentiation and the functional integration of newborn neurons into the pre-existing network. Astrocyte communication with neurogenic niche cells also greatly depends on their paracrine activity. As one of the main secretory cells of the CNS [19], astrocytes release a myriad of gliotransmitters, neuromodulators and morphogens as well as metabolic, trophic and neuroprotective factors [13,14], by which they finely and positively regulate multiple steps of the neurogenic process. On the other hand, astrocytes can negatively modulate neurogenesis by both cell–cell contact and paracrine activity [17].

From being “silent” in healthy brain, microglia active role in adult neurogenesis has been profoundly reassessed in recent years. Evidence indicates that activated microglia plays a Janus-faced role in the context of adult neurogenesis, by favouring or counteracting NSC proliferation, differentiation and survival of adult-born neurons. These actions are mediated by both direct contact and paracrine mechanisms. For example, microglia have been shown to phagocyte newborn cells that undergo apoptotic death in SGZ and SVZ, thus ensuring the homeostasis of the neurogenic process [20,21]. In addition, microglia act as antigen-presenting cells interacting with peripherally derived immune cells. This interaction mainly occurs in the SVZ that is highly vascularized [22,23], thereby influencing NSC final commitment toward neuronal or glial phenotypes depending on the different kinds of activating T-cell stimuli (e.g., IL-4 or INF-y) [24]. On the other hand, microglia can influence adult neurogenesis through secretion of proneurogenic and/or antineurogenic molecules, whose balance determines the net outcome of adult-born neurons [25]. In particular, as the main driver of inflammatory processes in the brain, cytokines released by microglia can dramatically affect adult neurogenesis [26]. Altogether, astrocyte and microglia plasticity—which is reflected by their ability to acquire an anti- or pro-neurogenic phenotype—M1- and M2-states for microglia [27] and A1- and A2-states for astrocytes [28]—make these cells crucial actors in influencing NSC as well as responding to the complex and continuously changing neurogenic niche microenvironment.

An underestimated actor of the adult neurogenic niche is the neuronal component, which can participate in the regulation of neurogenesis dynamics. Recent evidence indeed suggests a bidirectional communication between developmentally and adult-born neurons [29,30]. Additionally, mature neurons were proposed to modulate adult neurogenesis by sending chemical signals to NSC [31].

## 2. Extracellular Vesicles

### Biogenesis and Function

Extracellular Vesicles (EVs) are a heterogeneous population of membrane-bound entities that are released by both eukaryotic and prokaryotic cells [32] and that, by transporting different types of biomolecules, are key players in intercellular communication. Although much progress has been made in recent years in dissecting the molecular mechanisms underlying cargo packaging in recipient cells [33], further investigations are required to fully characterize the machineries and cellular pathways that determine the ultimate function (signaling or disposal) of cargo sorting in EVs.

**Figure 1:**
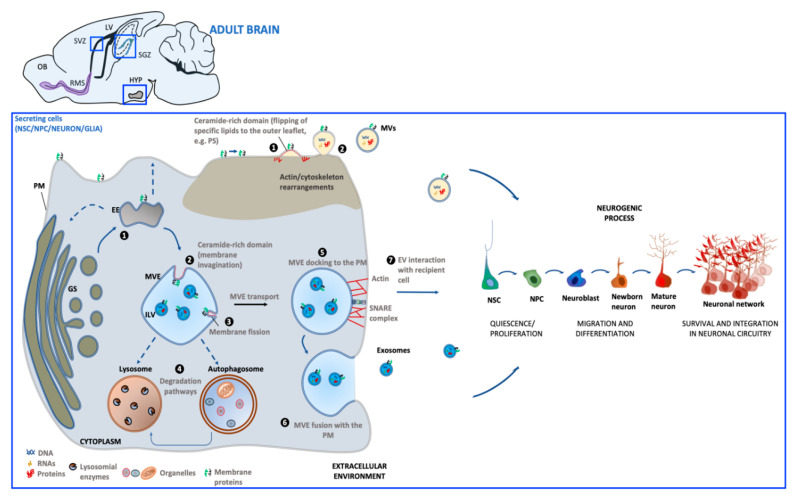
Schematic representation of extracellular vesicles’ (EV) biogenesis and release in the adult neurogenic niche. Biogenesis of microvesicles (MVs) (*brown background*) involves molecular machineries and membrane microdomains that promote the outward budding of the PM (1), followed by MV release in the extracellular environment (2). Exosome biogenesis (*blue background*) occurs upon maturation of early endosomes (EE) derived from the Golgi system (GS) into multivesicular endosomes (MVEs). Alternatively, EE can undergo retrograde transport to GS (*dashed arrows*) or recycling back to the PM (*dashed arrows*) (1). Exosomes are generated through membrane invagination of MVE (2), followed by ILV formation (3). Once matured, MVEs can be targeted to lysosomes/autophagosomes for cargo degradation (4), or be directed towards the PM (5). MVE fusion with PM (6) allows for exosome extracellular release (7). Both MV and exosome interaction with recipient cells can influence steps of the neurogenic process. [PM: plasma membrane; PS: phosphatidylserine; ILV: intraluminal vesicle; OB: olfactory bulb; SVZ: subventricular zone; SGZ: subgranular zone; HYP: hypothalamus; LV: lateral ventricle; RMS: rostral migratory system].

The generation of EVs requires the fine-tuning of several intracellular molecular machineries and trafficking processes (as schematized in Figure 1). The best-characterized EVs are exosomes and microvesicles (MVs). Although the biogenesis of exosomes and EVs occurs at distinct sites within the cell, some common intracellular pathways and sorting machineries are involved in the generation of both types of EVs, thus hindering the possibility of discriminating between the different vesicle subpopulations [34].

Exosomes (30–100 nm) derive from the endosomal compartment. Their formation starts with the generation of multivesicular endosomes (MVEs), spherical endosomes consisting of a limiting membrane and intraluminal vesicles (ILVs). The formation of MVEs is orchestrated by a complex of proteins called the endosomal sorting complex required for transport (ESCRT) which participates in the channeling of molecules into ILVs as well as the budding and fission of ILVs within MVEs [35]. However, there is evidence that exosome formation can also occur in a ESCRT-independent process [36]. MVE docking at the plasma membrane (PM) is regulated by RABs, actin and SNARE proteins, which finally promotes MVE fusion with PM and the release of the contained ILVs in the extracellular milieu as exosomes.

Microvesicles (MVs) (50–1000 nm) originate directly from PM by an outward budding which requires redistribution in lipid and protein composition and modifications in Ca^2+^ levels [37]. In MV biogenesis, Ca^2+^-dependent enzymes such as aminophospholipid translocases (flippases and floppases), scramblases and calpain drive the externalization of phosphatidylserine, which then drives changes in local membrane curvature and restructuring of the underlying actin cytoskeleton. These events are followed by the ATP-dependent fission process that leads to vesicle budding off from the PM and its subsequent release in the extracellular space [38,39].

Once released into the extracellular environment EV docking on target cell is regulated by specific interaction between membrane receptors on the recipient cell and EV enriched proteins. The uptake mode of EVs may be dependent on cell type, its physiological state as well as on the molecular composition at the PM of the target cell [40]. EVs can bound to the cell surface and initiate intracellular signaling pathways, be internalized or directly fuse with PM [41]. If internalized, EVs can fuse with the PM and release their contents into the cytoplasm of the recipient cell. Alternatively, EVs can target the endosomal pathway of the receiving cell and be directed toward the lysosome for the degradation of EV content to provide recipient cells with essential biological metabolites.

After interaction with target cells, EVs can elicit a variety of functional responses by delivering a wide array of biologically active molecules. These include lipids, proteins and nucleic acids, mRNA and other RNA species [(transfer RNA (tRNA), long non-coding RNA (lnRNA), micro RNA (miRNA), small nuclear RNA (snRNA), small nucleolar RNA (snoRNA)], which can be translated into proteins or regulate transcription in recipient cells, resulting in transient or persistent cellular phenotypic changes [42,43]. In particular, increasing evidence suggests that the effect of EVs on target cells is mainly dependent on the profile of intravesicular miRNA content [44]. By transferring miRNAs to target cells, EVs are now recognized as active players in intercellular gene regulation [45] because of their key natural roles in several cellular processes, including proliferation, differentiation, survival and apoptosis [46].

To date (October 2020), according to the compendium of molecular data Vesiclepedia, a total of 349,988 proteins, 27,646 mRNAs, 10,520 miRNAs and 639 lipids have been detected in exosomes, MVs and apoptotic bodies, suggesting a high degree of complexity in EV-mediated communication.

Importantly, the EV cargo is strictly dependent on the status of parental cells, making these biological entities critical in transmitting both physiological and pathological signals.

EVs can support normal physiology by affecting stem cell maintenance [42], tissue repair [47], immune response [48], and blood coagulation [49], lipid metabolism [50], synaptic plasticity [51].

Under pathological situations, EVs can transport disease-associated proteins [52], thus contributing to propagate detrimental signals. Finally, since several molecular constituents in EVs have been found to be associated with specific diseases and treatment responses, EVs may represent reliable biomarkers which could serve as a diagnostic tool [53].

## 3. Extracellular Vesicles Generated in Adult Neurogenic Niches

The first publication of EVs released by neural cells was in 2004, when Février and colleagues demonstrated that glial cell lines overexpressing a prion protein released EVs that were capable of transferring infectivity in vitro and in vivo [54]. This work paved the way for the study of EVs as new tools exploited by neural cells for communicating with each other to guarantee normal brain function.

More recently, it has become increasingly evident that EVs may represent an additional key component of intercellular neurogenic niche communication. NSCs, neurons and glia have all been reported to release EVs that, in turn, can mediate a generalized cross-talk by niche components. In the next paragraphs, we will summarize the current evidence on the emerging role of EV-based cross-talk in the direct or indirect modulation of adult neurogenesis.

NSC-, neuron-, astrocyte- and microglia-derived EVs will be analyzed in terms of cargo content and functional impact on neurogenesis. When data are available, we discussed the potential role of this peculiar form of intercellular communication in affecting different steps—proliferation, survival, fate specification, maturation and integration—of the complex cellular dynamics occurring in adult neurogenic niches.

### 3.1. NSC-Derived Extracellular Vesicles

Endogenous adult NSC can generate EVs (NSC-EV).

A large array of studies have suggested that the exogenous administration of NSC-EVs in relevant animal models of acute and chronic neurodegeneration can foster neuroprotection and neuroplasticity [55,56,57,58,59]. Interestingly, these in vivo beneficial effects might largely depend on EV’s intrinsic properties that contribute to re-creating an immune-permissive environment that promotes brain repair and neurogenesis. Surprisingly, as of today, a much more limited number of studies have directly focused on the molecular/functional characterization and on the endogenous role of NSC-EVs on neuroplasticity and neurogenesis.

Based on this lack of knowledge, herein we reviewed the current direct and indirect knowledge of how endogenous NSC-EVs may affect and modulate different cellular components of the adult niche. Although more experimental efforts are required in this field, some interesting studies have opened the way to an initial understanding of the endogenous NSC-EV cargo and function. The different classes of pro-, anti-neurogenic and glia modulatory molecules found in EVs derived from NSCs are summarized in Table 1.

#### 3.1.1. NSC-EVs: Effects on Adult NSC and Their Neuronal Progeny

The majority of studies addressing the physiological role of NSC-EVs in adult neurogenesis focused on their miRNA content. MiRNAs are key regulators of the multistep process of adult neurogenesis in adult SVZ and SGZ niches [97]. An insight into the complex and widespread action of NSC-EVs has been provided by next-generation sequencing of the RNA contents of human NPC-EVs. These efforts led to the identification of several exosomal miRNAs that were differently expressed compared to the cells, with roles in neural regeneration, neuroprotection, aging and neural plasticity [98,99]. Potentially interesting information on NSC-EV-associated miRNAs that may affect neurogenesis was also derived by comparison between primary NPC-EV and EVs produced by NPC obtained by direct conversion of somatic cells into induced NPC (iNPC). In vitro experiments showed that, unlike primary NPC-EVs, iNPC-EVs had no proneurogenic effects, while both EVs had no effect on glial differentiation [78]. By comparing the miRNA profile of primary NSC-EVs and iNSC-EVs, several differentially expressed miRNAs were identified, with miR-21a being highly enriched in primary cell-derived exosomes. Using miR-21a specific inhibitor and mimics, the authors demonstrated a key role of miR-21a in the in vitro generation of newborn neurons. Gene ontology analysis identified *Sox2* and *Stat3*, well known regulators of NPC proliferation and differentiation [100,101], as some of the target genes downregulated by miR-21a. Similarly, it has been suggested that miR-9 and miRlet-7b, which are upregulated and downregulated, respectively, in NPC-EVs compared to iNPC-EVs, may affect NSC fate. In particular, both miRNAs suppress expression of the orphan nuclear receptor TLX—a receptor known to maintain adult NSCs of both DG and SVZ in an undifferentiated, proliferative state [102].

Altogether, these findings suggest that miRNAs associated to NSC-EVs may be involved both in regulating stem cell quiescence/proliferation ratio and in cell-fate specification.

Modulation of NSC-EV miRNA cargo may also reflect how adult NSCs change their transcriptome in order to rewire the hippocampal circuit network through pro-survival and cell fate signalling. It has been found that upon treatment with kainic acid (KA), adult hippocampal NSCs upregulate the expression of miR-124 and miR-137 [82], which are known to target the pro-apoptotic protein BCL2L13 cooperatively and to regulate NSC activation/proliferation, fate specification and differentiation [81]. A few hours after KA insult, miR-137 was preferentially retained in the cellular fraction, whereas miR-124 was mostly sorted within NSC-EVs. At later times, NSC-EVs further increased their miR-124/miR-137 ratio, compared to parent cells. In consideration of its antiapoptotic function, the sorting of miR-124 in NSC-EVs may contribute to the maturation and survival of DG neurons, without affecting NSC proliferation [103].

The protein cargos of NSC-EVs may also be physiologically relevant. Characterizations of EV contents through proteomics analysis showed that NPC-EVs contained growth-factor-associated proteins that were predicted to activate the downstream extracellular signal-regulated kinase (ERK) pathways [60]. The treatment of NPCs with NSC-derived EVs was able to promote their proliferation in vitro. Both adult mouse and human NSCs have been shown to transport metabolic enzymes via EVs. Recently, Iraci and colleagues evaluated the ability of NSC-EVs to produce and consume metabolites [72]. Interestingly, asparagine was the highest consumed metabolite and aspartate/glutamate were the major released metabolites. The enzyme asparaginase-like protein 1 (Asrgl1) was identified as being responsible for EV-associated metabolic activity. The authors demonstrated that EVs do not acquire such metabolic function *de novo* but that L-asparaginase activity is transferred from NSC. Altogether, these data propose that EVs can function as independent metabolic units that are able to modify the concentration of critical nutrients in the extracellular milieu of the niche. For example, aspartate, released from NSC-EVs, may play a supportive role in cell bioenergetics in the neurogenic niche. Our full understanding of the unexpected ability of NSC to propagate, via EVs, specific metabolic signals to the surrounding cells in the neurogenic niche is just the beginning. Its key implications for the modulation of the adult neurogenesis process await future investigation.

Not only may NSC-EVs influence the neurogenic microenvironment with their specific cargo, but they may also affect it differently in a strictly context-dependent manner. It has been demonstrated that when NSCs are grown in cytokine-enriched media that may mimic a proinflammatory (Th1-like) or anti-inflammatory (Th2-like) microenvironment, EV RNA and protein cargo sorting is significantly modified [77].

#### 3.1.2. NSC-EVs: Effects on Glial Cells

The miRNA content of NSC-EVs has also been proposed as the main driver of phenotypic changes observed in recipient glial cells within the niche. By using both in vitro and in vivo models, it was demonstrated that SVZ NSC-EVs acted as microglia morphogens by activating a transcriptional program associated with immune and inflammatory responses [83]. RNA sequencing of NSC-EVs identified four highly enriched miRNAs, miR-9, miR-let-7, miR-26a, and miR-181c, with key roles in regulating microglia morphology and physiology [84,85,86,87]. Moreover miR-9 and miR-let-7 have documented roles in the regulation of adult neurogenesis [97]. In particular, miR-let-7 was found to recapitulate NPC-EV mediated increase in the number of CD11b^+^ microglia in the SVZ region as well as cytokine release by microglia in vitro [83]. Moreover, the injection of conditioned media derived from NPC-EV-treated microglia into the lateral ventricle reduced the proliferation of murine SVZ NPC, suggesting a NPC-microglia cross-talk that ultimately generates a negative feedback loop onto NPCs [83].

In addition to modulation, by miRNA delivery, of gene expression in target cells, NPC-EVs have been found to transport mRNAs and proteins related to IFN-γ signaling pathway. Cossetti and colleagues demonstrated that pro-inflammatory (Th1-like) stimulation of NPCs caused the release of NPC-EV-bound IFN-γ capable of activating signaling pathways in recipient cells [77]. These findings suggest the ability of EVs released by NSCs under inflammatory conditions to preferentially or selectively target astrocytes and microglia, given the largely documented evidence of IFN-γ signaling in both the cell types [75,76].

#### 3.1.3. NSC-EVs: Endocrine Functions

Interestingly, the EVs released by endogenous NSC may not only exert a direct, local effect on adult neurogenesis. NSC-EVs may also play a role as active physiological effectors in additional brain and systemic functions. A particularly interesting example of the physiological relevance of NSC-EVs at a more systemic level can be drawn from the adult neurogenic hypothalamic niche. Very elegant studies by Cai and colleagues have suggested that the hypothalamus plays a critical role in systemic aging [104]. The same authors also showed that in middle-aged mice there was a substantial loss in Hypothalamic NSC (HytNSC), and that ablation of these cells or their in vivo replenishment by transplantation resulted, respectively, in faster and slower ageing in mice [99].

Interestingly, HytNSC secreted exosomes that with their miRNA cargo contributed not only to local modulation in the niche but also to a pool of CSF circulating miRNAs [99]. Such miRNAs were heavily reduced in the CSF with aging. The effect of the direct release of EVs generated from postnatal hypothalamic NSCs into the brain of middle-aged mice was examined. Mice that were treated in the hypothalamic third ventricle with EVs derived from postnatal cultured HytNSCs displayed reduced age-related changes, including improved locomotion, coordination, novel object recognition (NOR) and sociality, in comparison to vehicle-treated mice. Intriguingly, the miRNA content of hypothalamic NSC-EVs were reported to partially mediate the anti-aging effect of hypothalamic NSCs. Since age-dependent loss of hypothalamic NSC correlates with aging-related physiological declines, the overall concentration of specific miRNAs coming from hypothalamic NSC-EVs decreases considerably, which may contribute to the process of aging. On the other hand, the transplantation of healthy hypothalamic NSCs into the aging brain maintained the concentration of miRNAs at optimal levels through the release of specific EVs, which leads to successful aging and lifespan extension. Interestingly, when exosome secretion was inhibited in the hypothalamic NSCs of young mice through lentiviral shRNA-mediated downregulation of Rab27a—a critical molecule involved in exosome secretion—such inhibition correlated with: i) downregulation of the age-specific pool of CSF miRNAs; ii) impairment in several aspects of age-related physiology at 6 weeks post-injection [99]. 

Altogether, these data underlie an “endocrine” function of HytNSCs which can partially control systemic and brain aging speed through NSC-EV miRNAs, rather than a local and neurogenesis-restricted mechanism, as previously corroborated by studies correlating the decline in adult neurogenesis with the advent of aging-associated disorders [105,106,107].

### 3.2. Neuron-Derived EVs in Neurogenic Niches

Neuron-derived EVs (NDEs) are increasingly gaining attention as a novel mechanism of cell-to-cell communication, including inter-neuronal crosstalk. Indeed NDEs can selectively bind to other neurons [108]. Based on these assumptions, within adult niches, NDEs can potentially contribute to modulation of neurogenesis by acting on NSC and/or their neuronal progeny directly or indirectly, via glial cells. Table 1 summarizes a list of pro-, anti-neurogenic and glia modulatory molecules associated with EVs of neuronal origin.

#### 3.2.1. NDE: Effects on NSC and Their Progeny

Several studies indicate that NDE release is regulated by cell depolarization, in an activity-dependent manner [109,110,111]. NDEs have been suggested to have a role in synapse elimination [69], the modulation of post-synaptic density and neuronal synaptic plasticity [71], regulation of brain vasculature integrity [112], transport of trophic and pro-neurogenic proteins [66], removal of proteins during stress [67], as well as receptor elimination at synapses undergoing plastic changes [68]. Interestingly, a functionally interplay between adult-born and developmentally born neurons has been described, including a bidirectional communication between developmentally and adult-born neurons [29,30] and between mature neurons and NSC [31]. Potentially, NDEs produced in neurogenic niches by both developmentally generated neurons and adult-born neuroblasts/neurons may also affect the dynamics of the multi-step neurogenic process at different stages.

NDEs released by neurons have demonstrated to be critical for protein removal necessary for neurite elongation [113], a critical step required for adult-generated neurons to become assembled into functional networks. To investigate the role of NDEs in neural circuit development and neurogenesis, Sharma and colleagues treated human primary neural cultures with EVs derived from human induced pluripotent stem cell (hiPSC)-derived neurons [64]. The authors found that the treatment with NDEs increased cell proliferation and neuronal fate in developing neural cultures. Neonatal mice receiving NDEs injection into the lateral ventricles exhibited increased cell proliferation in the GCL when compared to mice treated with NDEs previously digested with Proteinase K to cleave to EV surface proteins.

Additional data suggesting the ability of NDEs to affect adult neurogenesis come, indirectly, from the identity of some of their cargo components. One is Cystatin C [66], a cysteine proteinase inhibitor which can act as an autocrine/paracrine cofactor that cooperates with fibroblast growth factor 2 (FGF-2) to support its mitogenic activity on adult NPCs both in vitro and in vivo [65]. miRNAs detected in NDEs are also directly involved in regulating neuronal differentiation of NSC. For example, one miRNA found in NDEs is miR-34a [89], which has been identified as a main regulator of DCX [88], a microtubule-associated protein expressed by newly born postmitotic neuroblasts [114]. Another one is miR-124 [92], expressed in DCX^+^ neuroblasts but not in early-stage SVZ NSC or transit amplifying cells, and described as a neuronal fate determinant in the SVZ [90]. Cheng and colleagues suggested that miR-124 controls the temporal progression of neurogenesis in the adult SVZ by downregulating Sox9 [91]—a factor that regulates glial fate specification and controls the transcription of glial gene networks in the CNS—to permit neuronal differentiation [115].

#### 3.2.2. NDE: Effects on Glial Cells

Although the limited number of studies describing the interaction between NDEs and glia hinders a comprehensive interpretation of this cross-talk within the adult neurogenic niche, the immunomodulatory potential of neuron-derived EVs may be relevant to a better understanding of glia-mediated regulation of the neurogenic process. Men and colleagues demonstrated that NDEs contain a subset of miRNAs that is distinct from the miRNA profile of parent cells [93]. Interestingly, miR-124-3p, one of the miRNAs essential for cell commitment toward neurogenic lineage during development [116] and highly expressed in NDEs, can be specifically transferred to astrocytes. In this cell phenotype, miR-124-3p increased expression of the glutamate transporter GLT-1 through specific inhibition of GLT-1-targeting miRNAs. GLT-1 is critical to proper synaptic transmission by maintaining the extracellular glutamate below neurotoxic levels [95]. In addition, glutamate re-uptake via astrocytic GLT-1 has been reported to stimulate neuronal lineage selection and inhibit glial commitment in NSC-astrocyte co-cultures [94].

NDEs released by activated neurons have been found to promote microglia inflammatory polarization (the so-called M1 state) through miR-21-5p [96] and up-regulate pro-phagocytic complement component 3 (*C3*) gene [117]. Notably, complement signaling is crucial for microglia-mediated synapse pruning, which may drive the integration of new neurons into pre-existing circuits during adult neurogenesis [118]. Moreover, it has been demonstrated that NPCs and immature neurons express receptors for complement fragments C3a and C5a (C3aR and C5aR) and that mice lacking C3 signaling have reduced basal SVZ/SGZ neurogenesis compared to control mice [119].

### 3.3. Glia-Derived Extracellular Vesicles

Although the current knowledge on the role of glial-derived EVs in adult neurogenic niches is limited, growing evidence suggest that these biological entities may be major players in the communication of astrocytes and microglia with NSC and their progeny (for reviews, see [120,121]). In the following paragraphs, we will discuss the potential role of EVs derived from astrocytes [Astrocyte-Derived Extracellular Vesicles (ADEs)] and microglia [(Microglia-Derived Extracellular vesicles (MDEs)] in regulating the adult neurogenic process. In particular, we posit that glia-derived EVs may have a prominent role in regulating the dynamics in the neurogenic zones, based on their presence in the ADEs and MDEs of biomolecules that have been functionally characterized as modulators of adult neurogenesis (Table 2).

#### 3.3.1. EV-Associated Growth Factors

Several trophic factors have key roles in adult neurogenesis. Astrocytes have been found to release EVs containing the fibroblast growth factor-2 (FGF-2) and vascular endothelial growth factor (VEGF) [123], which have recognized roles in stimulating the proliferation and differentiation of NSCs in both SVZ and SGZ niches [174,175]. Fibroblast growth factor receptor 1 (FGFR1) is essential for the proliferation of NSCs in both adult neurogenic zones [122]. As far as VEGF is concerned, it exerts direct mitogenic effects on NSC via VEGFR-2/Flk-1 receptor activation [176,177]. In addition, extensive evidence supports VEGF’s crucial role in creating an angiogenic microenvironment that is permissive for newborn neuron integration. Palmer and colleagues reported that about a third of the dividing cells in the SGZ are endothelial precursors, which proliferate together with neural precursors, forming clusters which show strong positivity for VEGF and its Flk-1 receptor [61]. Moreover, VEGF has been shown to indirectly promote the neurogenic process by prompting endothelial cells to release BDNF which, in turn, regulates the survival and integration of newborn neurons [62].

#### 3.3.2. EV-Associated Enzymes and Transporters

Astrocyte reuptake of extracellular glutamate through membrane excitatory aminoacid transporters EAAT-1 and EAAT-2 is required for proper neurotransmission during normal brain function [95]. In addition, glutamate levels and receptors are key players at later stages of neuronal maturation in the hippocampus, when newly generated neurons are established in their final GCL position and start receiving abundant glutamatergic afferents [124]. The discovery that astrocytes release EVs containing EAAT-1 [127] suggests that these biological entities might serve as independent units regulating adult-born neuron integration in neuronal network and synaptic transmission. In particular, protein kinase C (PKC) activation in primary rat astrocyte cultures was found to cause a subcellular re-distribution of EAAT-1 from plasma membrane to the endosomal compartment and finally an enrichment in ADEs. In addition to EAAT-1, ADEs also contain α-Na/K-ATPase and glutamine synthetase which are essential, respectively, for the electrochemical plasmalemmal Na^+^ gradient required for amino-acid transport and for the conversion of glutamate into glutamine [127]. Based on these observations, ADEs might serve a functional role in extracellular glutamate elimination and, indirectly, they regulate levels of GABA, which is primarily synthesized from glutamate.

Purinergic signaling has been demonstrated to regulate adult neurogenesis in both SGZ and SVZ regions [178,179]. By carrying active ecto-enzymes nucleoside triphosphate diphosphohydrolases (NTPDases), which hydrolyze extracellular tri- and di-phosphate nucleotides to nucleoside monophosphates [131], ADEs may also be involved in the regulation of the proliferation and differentiation of adult NSCs. NTPDase2 has emerged as a main modulator of nucleotide signaling, regulating ATP bioavailability in the neurogenic niche and its subsequent interaction with NSC P2Y receptors. Indeed Gampe and colleagues demonstrated that, compared to wild-type mice, NSC proliferation was increased in both the SVZ and DG of NTPDase2 knockout animals [128]. Using pharmacological and genetic strategies, Cao and colleagues demonstrated that astrocytic ATP-mediated purinergic signaling was necessary and sufficient to stimulate NSC proliferation both in vivo and in vitro [180]. In line with this, adenosine, a metabolic product of ATP hydrolysis, was found to negatively affect hippocampal NSC proliferation [129] and to inhibit, in vitro, neuronal differentiation of SVZ NSCs via A1 receptor activation [130].

MDEs were found to carry active aminopeptidase CD13 [136]. This enzyme hydrolyzes leucine- and methionine-enkephalins, thus regulating ligand bioavailability for opioid receptors, which have been demonstrated to negatively regulate the proliferative and differentiative responses of adult NSCs [133,135] as well as their progeny survival [134].

MDEs were also found to display the monocarboxylate transporter 1 (MCT-1), together with enzymes necessary for anaerobic glycolysis or lactate production [136]. These observations suggest a novel route for lactate release that may serve as a supplementary energy substrate for neurons during synaptic activity. Interestingly, among the different mechanisms by which physical exercise enhances hippocampal adult neurogenesis, lactate transport to the CNS has been recently proposed [137]. Intraperitoneal injections of lactate in mice correlate with an increased number of newly generated neurons in the DG. Interestingly, lactate-induced increase in neurogenesis was not due to increased cell proliferation or increased neuroblast differentiation, but rather to an increased survival rate of newly generated mature neurons.

Overall, these studies suggest a potential role of ADEs and MDEs in finely regulating gliotransmitters and metabolites availability in the adult neurogenic niche, which, in turn, may affect proliferation, differentiation and survival rates of NSCs and their neuronal progeny.

#### 3.3.3. EV-Associated Neuroprotective Proteins

Glial-derived EVs have been reported to transport molecules with neuroprotective and regenerative activities. These molecules may also affect the rate of newborn neuron survival in the adult neurogenic niche.

ADEs were found to transport synapsins, phosphoproteins which have been shown to be released by ADE lumen and mediate protective effects on neuronal cultures exposed to oxygen-glucose deprivation (OGD) or oxidative stress [141]. Synapsins, well known for their role in synaptogenesis and neuronal plasticity [140,181], are also involved in the proliferation and survival of NSCs in adult DG [138].

Heat shock protein (HSP70), a molecular chaperone with documented neuroprotective function in several acute and chronic neurodegenerative disorders [182,183,184], is released by astrocytes through EVs [144]. Intraperitoneal administration of HSP70 improved novel object recognition in 8-week-old mice. This behavioural effect correlated with increased cell proliferation and neuroblast differentiation in the DG [143].

Neuroglobin (NGB) is a neuroprotective, anti-oxidant, anti-apoptotic, and anti-inflammatory protein [185,186,187] specifically detected in ADEs [146]. The protein has been implicated in neurogenic effects both in vitro and in vivo [145]. Lentivirus (Lv)-mediated overexpression of NGB in SVZ NSCs resulted in increased cell proliferation and neuronal differentiation via Wnt signaling. In vivo, intracerebroventricular (i.c.v.) injection of Lv expressing-NGB in a murine stroke model (MCAO) increased PSA-NCAM^+^ neuroblasts and Tuj1^+^ immature neurons in the SVZ and peri-infarct cortex compared to sham group [145].

#### 3.3.4. EV-Associated Cytokines

Evidence of both the detrimental and beneficial consequences of neuroinflammation within adult niches has been reported [188]. Such opposite effects can, at least in part, depend on different receptor-mediated pathways. As an example, TNF has been reported to inhibit or stimulate hippocampal neurogenesis via the activation of TNFR1 or TNFR2, respectively [159]. On the other hand, more consistent data indicate that anti-inflammatory mediators, including IL-10 and IL-4, positively modulate neurogenesis [189,190].

Although cytokines are generally thought to exert biologic influence as soluble molecules, their release in an EV-encapsulated form has been documented in different in vitro and in vivo biological systems [191]. For example, glial cells can release EVs which can carry TNF and IL-6 [158], IL-1β [151,152] and IL-4 [192]. The mechanisms underlying the sorting of a given cytokine within glia-derived EVs are strictly dependent on the nature of the stimulus and the cell type. ATP is a potent inducer of EV release by glial cells. Bianco and colleagues found that upon ATP activation of receptor P2X7 astrocytes released EVs containing IL-1β that were associated with lipid membrane rearrangements induced by rapid activation of acid sphingomyelinase [152]. Similarly, the activation of P2X7R in primary microglia by astrocyte-derived ATP caused the shedding of IL-1β-containing EVs from microglia surface [151], thus suggesting similar molecular mechanisms underlying EV release in response to the same stimulus.

In another study, astrocyte cultures were found to upregulate or downregulate inflammatory cytokines, such as IL-1β, IL-6 and TNF-α upon treatment with MDEs derived from ATP-treated or untreated microglia, respectively [193]. This suggests that EV content and activity reflects the functional state of the parental cell.

In addition to being conveyed by EVs within adult niches, cytokines have also been shown to dramatically modulate the proteomic signatures of both ADEs and MDEs, with diverse and profound effects on the regulation of synaptic activity and/or neuroplasticity. Antonucci and colleagues found that EVs derived from microglia treated with ATP enhanced spontaneous and evoked excitatory transmission in hippocampal neurons, as indicated by the increase in miniature excitatory postsynaptic current (mEPSC) frequency and amplitude of EPSCs [194]. In another study, quantitative mass spectrometry analysis of EVs derived from primary human astrocytes treated with IL-1β (IL-1β-ADEs) revealed 113 proteins that were uniquely expressed compared to ADEs derived from the control counterpart (CTRL-ADEs) [195]. The treatment of murine primary neurons with IL-1β-ADEs reduced neurite outgrowth and branching compared to CTRL-ADEs. Moreover, the analysis of neuronal firing by multi-electrode arrays evidenced that CTRL-ADEs accelerated neuronal maturation for firing, which was counteracted by IL-1β-ADE treatment. The distinctive ability of EVs to modulate synaptic activity might profoundly affect adult neurogenesis, by virtue of the critical role of inhibitory and excitatory input received by neuroblasts and young maturing cells during this process (for a review, see [196]).

Finally, the proteomic signature of glial-derived EVs can affect their potential to act locally in the neurogenic niche and/or propagate inflammatory signals outside the CNS, due to EV’s ability to cross the blood brain barrier (BBB) and activate the peripheral immune system. The proteomic profiling of ADEs derived from IL-10-treated primary rat cortical astrocytes identified a set of proteins primarily involved in neurite outgrowth, dendritic branching, regulation of synaptic transmission, and promoting neuronal survival, and therefore with potential positive effects on adult neurogenesis [197]. In contrast, IL-1β-ADEs were enriched with proteins that regulated peripheral immune response and immune cell trafficking to the CNS. This is in line with a recent report demonstrating that after i.c.v. injection, IL-1β-ADEs entered the peripheral circulatory system and induced up-regulation of liver pro-inflammatory cytokines, that, in turn, caused leukocyte activation and transmigration into the brain [198]. The ability of glial-derived EVs to activate peripheral immune cells deserves considerable attention, in virtue of the large amount of studies demonstrating a pivotal role of the infiltrating immune cells in the regulation of adult neurogenesis [199,200].

#### 3.3.5. EV-Associated miRNAs

As previously discussed, a vast array of studies reported that miRNAs play key roles in regulating NSC fate in the adult SVZ and SGZ [97]. Notably, several miRNAs have been detected in EVs derived from astrocytes and microglia. Table 2 summarizes selected literature reports of miRNAs whose presence in ADE and MDE has been demonstrated as well as their potential effect on different steps and cellular types participating in adult neurogenesis. Interestingly, once again, miRNAs detected in glia-derived EVs show a substantially distinct expression profile compared to their parental cells, suggesting a unique repertoire of ADE- and MDE-associated miRNAs that might potentially contribute to regulating adult neurogenesis under physiological conditions. Cooperation among miRNAs from ADEs and MDEs for the modulation of adult neurogenesis can also be envisioned. ADE and MDE miRNA cargos have been shown to be regulated by external stimuli, similarly to the extensive modulation of adult neurogenesis in response to environmental conditions and experiences. In a recent study, early exposure of mice (P21-60) to high-fat diet (HFD) was found to be associated with reduced adult hippocampal neurogenesis, as suggested by decreased SGZ cell proliferation and a reduced number of DCX^+^ neuroblasts in parallel with hippocampal inflammation [201]. In vitro stimulation of primary microglia with palmitic acid (PA), the most common saturated fatty acid in western diets and in HFD, could reproduce a pro-inflammatory phenotype. At least some of its negative in vitro effects on primary neuronal cells were reproduced by EVs derived from PA-stimulated microglia [201]. Notably, PA can also negatively affect the survival and neuronal differentiation of hippocampal NSC [202].

Future studies specifically addressing their role in the modulation of NSC proliferation and fate specification are certainly needed to further understand the pleiotropic effects of glia-derived EVs on neuroplasticity.

## 4. Conclusions and Future Perspectives

Extracellular vesicles represent a relatively new mechanism for intercellular communication, allowing cells to exchange any type of biological molecules, ranging from proteins to nucleic acids and lipids. The interaction of EVs with recipient cells can not only be specifically targeted but also generate, in any given cell target, diverse effects, from activating signalling pathways to providing trophic support, depending on the functional state of the secreting cell, the mode of interaction, and the fate of the released EV.

**Figure 2:**
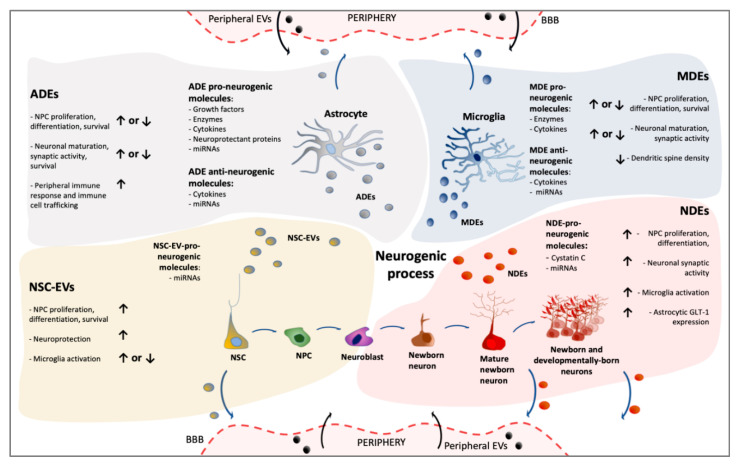
Different layers of complexity in EV-based signalling within neurogenic niches. Schematic representation of the potential impact of EVs generated by distinct cellular components of the niche (NSCs, astrocytes, microglia, neurons) on key steps of the neurogenic process. The nature of the pro- and anti-neurogenic EV-associated molecules is also summarized. Given their ability to cross the blood-brain barrier, EVs derived from niche cells can also exert effects in periphery. Similarly, peripherally generated EVs can reach the adult niche and potentially modulate neurogenesis. (EVs: extracellular vesicles NSC-EVs: neural stem cell-derived EVs; NDEs: neuron-derived EVs; ADEs: astrocyte-derived EVs; MDEs: microglia-derived EVs; BBB: Blood–Brain Barrier; GLT-1: glutamate transporter 1; ↑: increased; ↓: decreased).

In this review, we specifically focused on the potential role of EVs in the regulation of the adult neurogenic niche. Although the field is still in its infancy, NSCs, neurons and glia have all been reported to release EVs that, in turn, can mediate a generalized and complex crosstalk among niche components (Figure 2).

In recent years, EVs have been shown or proposed to actively influence the adult neurogenic process, either directly, by regulating NSC quiescence/proliferation/differentiation, migration, maturation, survival of neuroblasts and new-born neurons, or indirectly, for example via the modulation of the pro- and/or anti-neurogenic properties of glial cells. Surprisingly, although the critical role of astrocytes and microglia in the context of adult neurogenesis is well recognized, direct evidence concerning the impact of astrocyte- and microglia-derived EVs on cellular dynamics within the adult niche is still very fragmentary. Even more surprisingly, despite the vast array of studies using exogenous administration of NSC-derived EVs as a therapeutic strategy in preclinical models of CNS disorders, a much more limited amount of experimental efforts focused on the endogenous, physiological role of EVs on neuroplasticity and neurogenesis.

Although we did not focus our attention on these aspects, of course, other niche cells, including oligodendrocyte precursors and endothelial cells, which have been reported to release EVs capable of modulating neuronal properties [203,204], may well contribute to adult neurogenesis modulation. Similarly, given their ability to cross the BBB, EVs originating from cells located outside the brain can reach the adult niche and should be included in the overall picture. Conversely, as some studies have elegantly suggested, EVs produced in adult niches may have an impact systemically, for example on physiological aging [99].

Altogether, we believe that future work should better investigate the nature and the functional role of critical pro- and anti-neurogenic factors carried by extracellular vesicles within niches. Such studies have the potential not only to increase our current knowledge on the physiological role of EVs in neuroplasticity and in aging, but they may disclose novel pathophysiological pathways in CNS disorders. Since alterations of adult neurogenesis appear to be a common hallmark of different neurodegenerative diseases [205], understanding which and how factors drive changes in the cargo of EVs derived from niche cells could be useful for the design of early therapeutic interventions to face aberrant neuroplasticity in these disorders. A constantly growing branch in the EV field is that concerning their use as drug delivery system. In fact, in virtue of their natural cell-targeting abilities, biodistribution profiles, pharmacokinetics, low immunogenicity and intrinsic ability to cross tissue and cellular barriers, EVs are promising for therapeutic purposes targeting brain diseases. Pioneering bioengineering studies have suggested that it is possible to obtain EVs loaded with the desired cargo and functionalized with surface molecules of interest, to enhance cell targeting [206]. Last but not least, new promising avenues regard EVs clinical use as biomarkers [53,207,208], since they can be easily collected from blood, urine [209,210,211], and also CSF [212].

## Figures and Tables

**Table 1:** List of different classes of pro-, anti-neurogenic and glia modulatory molecules found in extracellular vesicles derived from neural stem/progenitor cells-(NSC-EVs) and neurons (NDEs).

Class of Molecule	Molecules	Cellular Process/Molecular Target	EV Type
Growth factors	Growth factor receptor cysteine-rich domain, EGF-like domain, EGF-like calcium-binding domain	↑ NSC proliferation by activating the down-stream extracellular signal-regulated kinase (ERK) pathways [60]	NSC-EVs [60]
	VEGF	↑ NSC proliferation in SGZ [61]; ↑ survival and integration of newborn neurons in the forebrain [62]	NSC-EVs [63]
Proteins	Flotillin, GAP43, Cadherin 2 L1CAM	Regulate NSC proliferation and neuronal differentiation [64]	NDEs [64]
	Cystatin C	↑ NSC proliferation by cooperating with FGF-2 [65]	NDEs [66]
	Ndfip1	↑ Removal of protein during stress [67]	NDEs [67]
	Synaptotagmin 4	↑ Retrograde signaling in pre-synaptic cells by releasing Syt4-bound exosomes [68]	NDEs [68]
	PRR7	↑ Removal of excitatory synapses by acting as a Wnt inhibitor [69]	NDEs [69]
	MAP1b	↑ synaptic transmission and plasticity [70]	NDEs [71]
Enzymes	Asrgl1	↑ levels of aspartate/glutamate [72] which regulate adult neurogenesis [73,74]	NSC-EVs [72]
Cytokines	INFγ	Regulate function of microglia and astrocytes by activating Stat1 in target cells [75,76]	NSC-EVs [77]
miRNAs	miR-21a	↑ NSC proliferation by targeting Sox2 and Stat3 [78]	NSC-EVs [78]
	miR-9	↓ NSC proliferation and ↑ neural differentiation by targeting the stem cell regulator TLX [79]	NSC-EVs [78]
	miR-let-7b	↓ NSC proliferation and ↑ neural differentiation by targeting the stem cell regulator TLX and the cell cycle regulator cyclin D1 [80]	NSC-EVs [78]
	miR-124 miR-137	Regulate NSC activation/proliferation, fate specification and differentiation by cooperatively targeting the pro-apoptotic protein BCL2L13 [81]	NSC-EVs [82]
	miR-let-7	Regulate microglia activation which negatively affect NSC proliferation in SVZ [83]	NSC-EVs [83]
	miR-9, miR-let-7, miR-26a, and miR-181c	Regulate microglia morphology and physiology [84,85,86,87]	NSC-EVs [83]
	miR-34a	Regulate NSC proliferation and morphology and function of newborn neurons by interacting with DCX [88] Target genes linked to the regulation of neuronal excitability, mitochondria oxidative phosphorylation, glycolysis, and resting state functional connectivity [89]	NDEs [89]
	miR-124	↑ NSC neuronal differentiation in SVZ [90] ↑ NSC neuronal differentiation in SVZ by targeting SOX9 [91]	NDEs [92]
	miR-124-3p	↑ GLT-1 expression in astrocytes [93] which ↑ NSC differentiation in vitro [94] and regulate synaptic transmission [95]	NDEs [93]
	miR-21-5p	↑ M1 polarization in microglia [96]	NDEs [96]

EGF: epidermal growth factor; VEGF: vascular endothelial growth factor; GAP43: growth-associated protein 43; L1CAM: L1 cell adhesion molecule; Ndfip1: Nedd4 family-interacting protein 1; MAP1b: microtubule -associated protein 1b; Proline-rich protein 7 (PRR7); Asrgl1: asparaginase-like protein 1; STAT1/3: signal transducer and activator of transcription 1/3; INFγ: interferon-γ. ↑: increased; ↓: decreased.

**Table 2:** List of different classes of pro- or anti-neurogenic molecules found in astrocyte-derived (ADEs) and/or microglia-derived (MDEs) extracellular vesicles.

Class of Molecule	Molecules	Cellular Process/Molecular Target	Glial EV Type
Growth Factor	FGF-2	↑ NSC proliferation and differentiation in SGZ and SVZ [122]	ADEs [123]
	VEGF	↑ NSC proliferation in SGZ [61]; ↑ survival and integration of newborn neurons in the forebrain [62]	ADEs [123]
Enzymes	EAAT-1	↑ NSC differentiation, maturation and integration of newly formed neurons in synaptic network in SGZ and SVZ through regulation of extracellular glutamate [124] and GABA [125,126] levels	ADEs [127]
	NTPDases	↓ NSC proliferation in SGZ and SVZ by regulating nucleotide ATP and adenosine levels [128] ↓ NSC proliferation in hippocampus [129] and in vitro neuronal differentiation of SVZ NSCs [130] through adenosine production	ADEs [131]
	CD13	↑ NSC proliferation, differentiation and survival through regulation of cAMP levels [132,133,134,135]	MDEs [136]
	MCT-1	↑ NSC survival of newly generated neurons [137]	MDEs [136]
Neuroprotectant proteins	Synapsins	↑ NSC proliferation and survival in adult DG [138] ↑ synapse development [139], neurotransmitter release [140], neurite outgrowth after oxygen-glucose deprivation (OGD)/oxidative stress [141]	ADEs [141]
	HSP70	↑ expression of genes involved in neuronal differentiation, synaptic activity, regulation of neuronal synaptic plasticity in Alzheimer’s disease [142] ↑ NSC proliferation, differentiation in DG via enhanced CREB phosphorylation and improve novel object recognition in mice [143]	ADEs [144]
	Neuroglobin	↑ NSC proliferation and differentiation in SVZ via Wnt signaling in murine stroke model [145]	ADEs [146]
Cytokines	IL-1β	↓ neurogenesis in DG by reducing the number of DCX^+^ cells [147] ↓ neurogenesis in DG by reducing the number of Nestin^+^ cells [148] ↓ hippocampal NSC proliferation in vitro via the nuclear factor-κB signaling pathway [149] ↑ NSC proliferation and differentiation through the activation of SAPK/JNK pathway [150]	MDEs [151], ADEs [152]
	IL-6	↓ DG NSC proliferation in vitro [153] ↓ NSC proliferation, differentiation and survival in DG [154] ↑ NSC self-renewal and maintenance in SVZ [155] ↑ NSC proliferation and neuronal maturation in SVZ and SGZ [156]	ADEs [157], MDEs [158]
	TNFα	↑ NSC proliferation and survival through TNFR2 in vitro and in vivo [159] ↓ NSC proliferation and ↑ cell death through TNFR1 in vitro and in vivo [159,160]	MDEs [158]
miRNAs	miR-302	↑ NSC proliferation, differentiation, survival through Cyclin D1/D2 and Fgf15 [161]	ADEs [162]
	miR-let-7d, miR-let-7a	↓ NSC proliferation and ↑ neural differentiation by targeting *TLX* receptor gene [163] ↑ NSC dopaminergic differentiation in olfactory bulb by *PAX6* targeting (miR-let-7a, [164])	ADEs [163]
	miR-145	↑ NSC differentiation through Sox2-Lin28/let-7 signaling pathway [165]	ADEs [163]
	miR-146a-5p	↓ NSC neural specification and synaptogenesis by targeting neuroligin 1 (*Nlg1*) and synaptotagmin 1 (*Syt1*) [166]	MDEs [167]
	miR-9	↓ NSC proliferation, ↑ NSC neural differentiation by targeting *TLX* receptor [79]	ADEs [168]
	miR-9, miR-124	↑NSC neural differentiation and dendritic branching of differentiated neurons by targeting the small GTP-binding protein Rap2a [169]	ADEs [168], MDEs [170]
	miR-184	↑ NSC proliferation, ↓ differentiation in SGZ by targeting *Numblike* [171]	ADEs [162]
	miR-34a	↑ NSC proliferation, ↓ dendrite branching and neuronal maturation by targeting *DCX* [88]	ADEs [172], MDEs [167]
	miR-106b, miR-93, miR-25	↑ NSC proliferation and differentiation toward neuronal lineage in vitro through insulin/IGF-FoxO pathway [173]	ADEs [162]

FGF-2: fibroblast growth factor 2; VEGF: vascular endothelial growth factor; EAAT-1: excitatory amino acid transporter 1; NTPDases: nucleoside triphosphate diphosphohydrolases; CD13: aminopeptidase N; MCT-1: Monocarboxylate transporter 1; CREB: cAMP response element-binding protein; HSP70: heat shock protein 70; SAPK/JNK: stress-activated protein kinases (SAPK)/Jun amino-terminal kinases (JNK); TNFR1/2: tumor necrosis factor receptor 1/2; IL-1β: interleukin-1β; IL-6: interleukin-6: TNFα: tumor necrosis factor α. ↑: increased; ↓: decreased.
